# NEDD4-2 and the CLC-2 channel regulate neuronal excitability in the pathogenesis of mesial temporal lobe epilepsy

**DOI:** 10.1038/s41598-024-52399-4

**Published:** 2024-02-28

**Authors:** Yuting Liu, Haiyan Yang, Rongrong Zeng, Lu He, Ting Xiao, Xiaomei Peng, Zhuo Kuang, Liwen Wu

**Affiliations:** 1 Pediatrics Research Institute of Hunan Province, Hunan Children’s Hospital, Changsha, Hunan China; 2 Department of Neurology, Hunan Children’s Hospital, Changsha, 410008 Hunan China; 3 Xiangya Hospital, Central South University, Changsha, Hunan China

**Keywords:** Cell biology, Neuroscience

## Abstract

An increasing number of studies have focused on the role of **NEDD4-2** in regulating neuronal excitability and the mechanism of epilepsy. However, the exact mechanism has not yet been elucidated. Here, we explored the roles of **NEDD4-2** and the **CLC-2** channel in regulating neuronal excitability and mesial temporal lobe epilepsy (MTLE) pathogenesis. First, chronic MTLE models were induced by lithium-pilocarpine in developmental rats. Coimmunoprecipitation analysis revealed that the interaction between CLC-2 and **NEDD4-2**. Western blot analyses indicated that **NEDD4-2** expression was downregulated, while phosphorylated (P-) **NEDD4-2** and **CLC-2** expression was upregulated in adult MTLE rats. Then, the primary hippocampal neuronal cells were isolated and cultured, and the **NEDD4-2** was knocked down by shRNA vector, resulting in decreased protein levels of CLC-2. While CLC-2 absence caused increased **NEDD4-2** in cells. Next, in an epileptic cell model induced by a Mg^2+^-free culture, whole-cell current-clamp recording demonstrated that **NEDD4-2** deficiency inhibited the spontaneous action potentials of cells, and CLC-2 absence caused more significant decrease in the spontaneous action potentials of cells. In conclusion, we herein revealed that **NEDD4-2** regulates the expression of CLC-2, which is involved in neuronal excitability, and participates in the pathogenesis of MTLE.

## Introduction

Mesial temporal lobe epilepsy (MTLE) is a group of diseases characterized by recurrent seizures and impaired learning and memory function, and hippocampal sclerosis is a prominent clinicopathological feature^1^. The pathogenesis of these diseases is related to the degeneration of hippocampal neurons, glial hyperplasia, abnormal loop remodelling and excitation-inhibition imbalance in the brain^2,3^. MTLE, the most common epilepsy, is generally intractable and accompanied with complicated clinical symptoms. Thus, studying the pathogenesis of MTLE is important for better clinical management and improvement of patient prognoses. Most recent studies have mainly focused on genetics factors, ion channels, inflammation and oxidative stress injury^4–6^. Nevertheless, the specific pathogenesis of MTLE has not been elucidated thus far.

**NEDD4-2** belongs to the Nedd4 (neural precursor cell-expressed developmentally downregulated gene 4) family, which is characterized by a C2 domain, 2–4 WW domains, and a HECT-type ligase domain, regulating several channels and receptors that affect neuronal excitability^7^. For instance, researchers have showed NEDD4-2 regulates the endocytosis of ion channels on the cell surface and recognizes and recovers ion channels^8^. In a study by Kabra et al.^9^, inhibiting the activity of NEDD4-2 significantly decreased the endocytosis and degradation of the epithelial Na^+^ channel ENaC, whereas NEDD4-2 overexpression increased the endocytosis and degradation of this channel. Therefore, NEDD4-2 may regulate the expression of ion channel proteins on the cell surface and their distribution in the cytoplasm/cell membrane. Selective deletion of *Nedd4-2* in mice caused elevated seizure susceptibility through ubiquitination AMPAR^9^; and conditional knockout of *Nedd4-2* in brain of mice reduced excitatory synaptic strength through ubiquitination of GluA1^10^. Also, *Nedd4-2* haploinsufficiency in mice increased susceptibility of seizures.Moreover, at least three missense mutations of *Nedd4-2* have been identified in epileptic patients^11–13^ Therefore, *Nedd4-2* is considered as an epilepsy-associated gene, with mechanisms needing further investigation. In our previous research^14^, we found that *Nedd4-2* is a gene encoding for a key E3 ligase involved in the pathogenesis of MTLE, but the downstream substrate of NEDD4-2 and the exact pathway remain unknown. Thus, whether NEDD4-2 regulates abnormal epileptiform discharges in MTLE by affecting the expression and distribution of ion channels on the cell surface is worthy of further exploration.

NEDD4-2 interacts with multiple members of the chloride channel family and regulates their distribution, activity and function^15^. One known family of intracellular chloride channel genes is CLC, which was first cloned from the electric organ of Torpedo marmorata by Jenstch et al.^16^. As a member of the CLC family, CLC-2 is also a target protein of NEDD4-2 and is widely distributed in the gastrointestinal tract, heart, kidney, and other tissues and organs, with relatively high expression in the brain^17^. CLC-2 expression in the brain differs significantly across regions and cell types; For example, CLC-2 is mainly expressed in the pyramidal cells in the CA1 and CA3 regions of the hippocampus, while in the cerebellum, it is primarily expressed in Purkinje neurons but hardly expressed in glial cells^18^. Based on the above findings, we propose a series of questions: Does CLC-2 expression differ in MTLE? Is CLC-2 expression in MTLE regulated by NEDD4-2? Further experiments are needed to answer these questions. Here, we explored the pathogenesis of MTLE as well as the regulation of NEDD4-2 and the CLC-2 channel from a new perspective.

## Materials and methods

### Animals and epilepsy induction

Twenty-one-day-old (which is similar to the onset age of MTLE patients) Sprague-Dawley rats weighing 30–50 g (clean grade, provided by the center for Experimental Animals, Central South University) were studied. They were housed in a ventilated quiet environment at room temperature (RT; 18–25 ℃) with 50–60% relative humidity and artificial circadian circulation lighting (Laboratory Animals Department, Central South University). The rats were fed nutritious food, and each cage contained only 4 rats to ensure that they could feed and drink on their own. The rats were allowed to adapt to laboratory conditions for at least 3 days before the experiments were begun. All animal procedures and protocols were approved by the Animal Ethics Committee of Hunan Children’s Hospital and followed the guidelines of the Institutional Animal Care and Use Committees of Hunan Children’s Hospital (Changsha, Hunan, China).

Seventy-five rats were randomly divided into two groups: a control group and a model group. The model rats were first injected with lithium chloride (127 mg/kg, i.p. Sigma-Aldrich Inc.), followed by an injection of pilocarpine hydrochloride (50 mg/kg, i.p. Sigma-Aldrich Inc.) 18–20 h later to induce status epilepticus (SE). Chloral hydrate (350 mg/kg, i.p. the pharmacy department of Xiangya Hospital) was administered 40 min after the onset of SE to terminate the seizure. The rats in which SE was not induced were removed from the experiment. Following pilocarpine treatment, the rats were video monitored for 8 weeks. All rats were divided into 6 groups based on the time course after epilepsy outbreak: the acute control (AC) group (normal control rats 2 h after saline administration), acute seizure (AS) group (seizure-induced rats 2 h after pilocarpine administration), latent control (LC) group (normal control rats 3 weeks after saline administration), latent seizure (LS) group (seizure-induced rats 3 weeks after pilocarpine administration), chronic control (CC) group (normal control rats 8 weeks after saline administration), and chronic seizure (CS) group (seizure-induced rats 8 weeks after pilocarpine administration). Behavioural observation and electroencephalogram (EEG) monitoring were carried out for each group of rats (n ≥ 3). For EEG monitoring, we followed a protocol established by Pitsch et al.^19^ with an improved electrode position. To monitor brain activity, we inserted three electrodes into the rat brain by drilling holes into the skull. Using a stereotax (Shenzhen Ryward Company), we placed the first electrode 2 cm in front-left of the front fontanelle and the second electrode 2 cm infront-right of the front fontanelle. The third electrode was placed 2 cm behind the posterior fontanelle. EEG monitoring was performed one week after electrode implantation using the RM6240 series multichannel physiological signal acquisition and processing system (Chengdu Instrument Factory) with the following parameters: scanning speed of 250 ms/div, sensitivity of 100 uv, time constant of 0.2 s, and filter frequency of 10 Hz.

### Morphological observation

The rats subjected to pilocarpine-induced SE were perfused at 8 weeks after pilocarpine-induced SE and processed for Nissl and Timm staining using a previously documented procedure routinely performed in our laboratory^20^. The rats were deeply anaesthetized with thionembutal and sequentially perfused through the heart. Frozen coronal sections were then processed for Nissl staining (6 µm) and Timm staining (30 µm). Nissl staining, using Cresyl violet as the Nissl stain substance, was applied to observe the extent of neuron loss. Timm staining was used to label the zinc-rich axon terminals of granule cells.

### Co-immunoprecipitation (Co-IP) assay

Co-IP assays were performed to examine the interaction between NEDD4-2 and CLC-2 according to the manufacturer's protocol, and complexes were precipitated with protein A/G agarose (Bimake). Then, complexes were subjected to western blot analysis using anti-NEDD4-2 (Abcam), anti-CLC-2 (Abcam).

### Western blotting

An individual protein sample from each group was chosen randomly for western blot analysis. Briefly, 30 μg of total protein from each sample was separated by 10% SDS-PAGE and transferred to PVDF membranes. The PVDF membranes were incubated overnight at 4 °C with monoclonal antibodies against NEDD4-2 (Abcam, 1:1000), P-NEDD4-2 (Abcam, 1:3000), and CLC-2 (Abcam, 1:1000), followed by incubation with HRP-conjugated sheep anti-rabbit IgG at a 1:2000 dilution. β-Actin (1:10,000) was used as an internal reference. The reactions were detected using an enhanced chemiluminescence (ECL) detection system. Signals on the blots were visualized by autography, digitally scanned and then quantified using Image J software. The experiment was repeated at least 3 times.

### Primary hippocampal neuron culture and establishment of an epileptic cell model

Neonatal Sprague-Dawley rats born within 24 h were taken for primary cell culture using a previously documented procedure routinely performed in our laboratory^20^. After routine sterilization and decapitation, the brain was removed, the bilateral hippocampus was separated, and the surface vessels were removed. Then, tissues were cut into small pieces and kept in a centrifuge tube; 2 mg/ml papain was added for digestion, and the reaction was terminated 15 min later with 10% fetal bovine serum (FBS). The sample was centrifuged for 5 min at 1500 r/min. The supernatant was discarded, and the pellet was washed and resuspended in implant solution. A few cell suspensions were used for trypan blue staining, and the cell density was adjusted to 5 × 10^5^/ml. The cells were inoculated in a 6-well culture plate (precoated with 0.1 g/L polylysine). After 4 h of culture, the same amount of media were replaced with maintenance solution, and the media was then changed every 4 days. The cell growth was monitored under an inverted phase contrast microscope. After 6 days of culture, the hippocampal neurons were used for subsequent experiments. Hippocampal neurons were then exposed to recording solution without MgCl_2_ to create a Mg^2+^-free culture. Three hours later, the in vitro hippocampal neuron cell model of epilepsy was acquired.

### Plasmid construction and neuron transfection

NEDD4-2-shRNA (GenBank accession number: NM_001008300) and CLC-2-shRNA (GenBank accession number: NM_017137) were purchased from Shanghai GeneChem Co., Ltd. Plasmid transfection was performed according to the method described by Li Zhang et al.^21^. Briefly, primary hippocampal neurons were randomly divided into a control group, an empty plasmid group, a NEDD4-2-shRNA group and a CLC-2-shRNA group. Twenty-four hours before transfection, the cell density of the neurons was adjusted to 1.2 × 10^7^/20 ml, and the cells were cultured in 15 cm cell culture dishes at 37 ℃ and 5% CO_2_. When the cell density reached 70–80% at 24 h, the cells were used for transfection. shRNA was transfected into neurons with ViraPower packaging mix using 100 µL of Lipofectamine 2000 reagent according to the manufacturer’s instructions. The viral supernatant was harvested 48 h after transfection, passed through a 0.45 µm filter, and concentrated, and the viral titre was determined. Seventy-two hours after plasmid transfection, fluorescence microscopy was used to observe the transfection efficiency of neurons, while the expression levels of NEDD4-2 and CLC-2 were determined by western blot.

### Detection of neuronal discharge by patch-clamp electrophysiology after plasmid transfection

Electrophysiological studies were carried out using patch-clamp techniques after neurons were transfected with the corresponding plasmids using the method described by Zhaohua Xiao et al.^22^. To record the recurrent spontaneous discharge of neurons, the cells were continuously monitored by patch-clamp electrodes utilizing the whole-cell current-clamp recording model. The recording solution of the treated epileptic cells contained the following (in mM): 145 NaCl, 2.5 KCl, 2 CaCl_2_, 10 HEPES, 10 glucose, and 0.001 glycine (pH 7.3); the osmolarity was adjusted to 325 mOsm with sucrose. Controls were maintained in regular recording solution^23,24^. The recording pipettes were made from glass capillaries (thickness = 0.225 mm) using a PC-10 puller (NARISHIGE). The pipet resistance was controlled at 1.5–2.0 MΩ by adjusting the pulling temperature. The pipet solution contained the following (in mM): 130 KCl, 2.0 MgCl_2_, 10.0 HEPES, 5.0 Na_2_-ATP, and 10.0 EGTA (pH 7.2–7.4); the osmolarity was adjusted to 290–295 mOsm with sucrose. The coverslips were placed in 35 mm dishes on the stage of an inverted microscope (Axiovert135, Zeiss, Oberkochen, Germany). For current recording, an EPC-10 USB Patch Clamp Amplifier (HEKA Elektronik) and Pulse software were used. All recordings were performed in the current-clamp recording mode at RT, and we monitored the membrane action potentials of every group continuously. Data were acquired by PatchMaster software (HEKA Elektronik) and analysed by Igo Pro6.10A, Excel 2010, SigmaPlot 10.0 and Origin Pro 8 software.

### Statistical analysis

The data in this study are presented as the mean ± standard deviation (SD) of at least three independent experiments. Statistical analyses were carried out with Student's t test for comparison of two independent treatments. Values were considered significant when p < 0.05. The statistical programme GraphPad Prism 7.00 (GraphPad Software, Inc., USA) was used to perform all the analyses.

### Ethical approval

This study, including the use of animals, was carried out in accordance with the recommendations of the Guide for the Central Laboratory of Hunan Children’s hospital. All procedures were approved by the Institutional Animal Care and Use Committee of Hunan Children’s hospital. And the study is in accordance with ARRIVE guidelines. This article does not contain any studies involving human participants that were performed by any of the authors.

## Results

### Abnormal behaviour and EEG in the rat model

**Figure 1:**
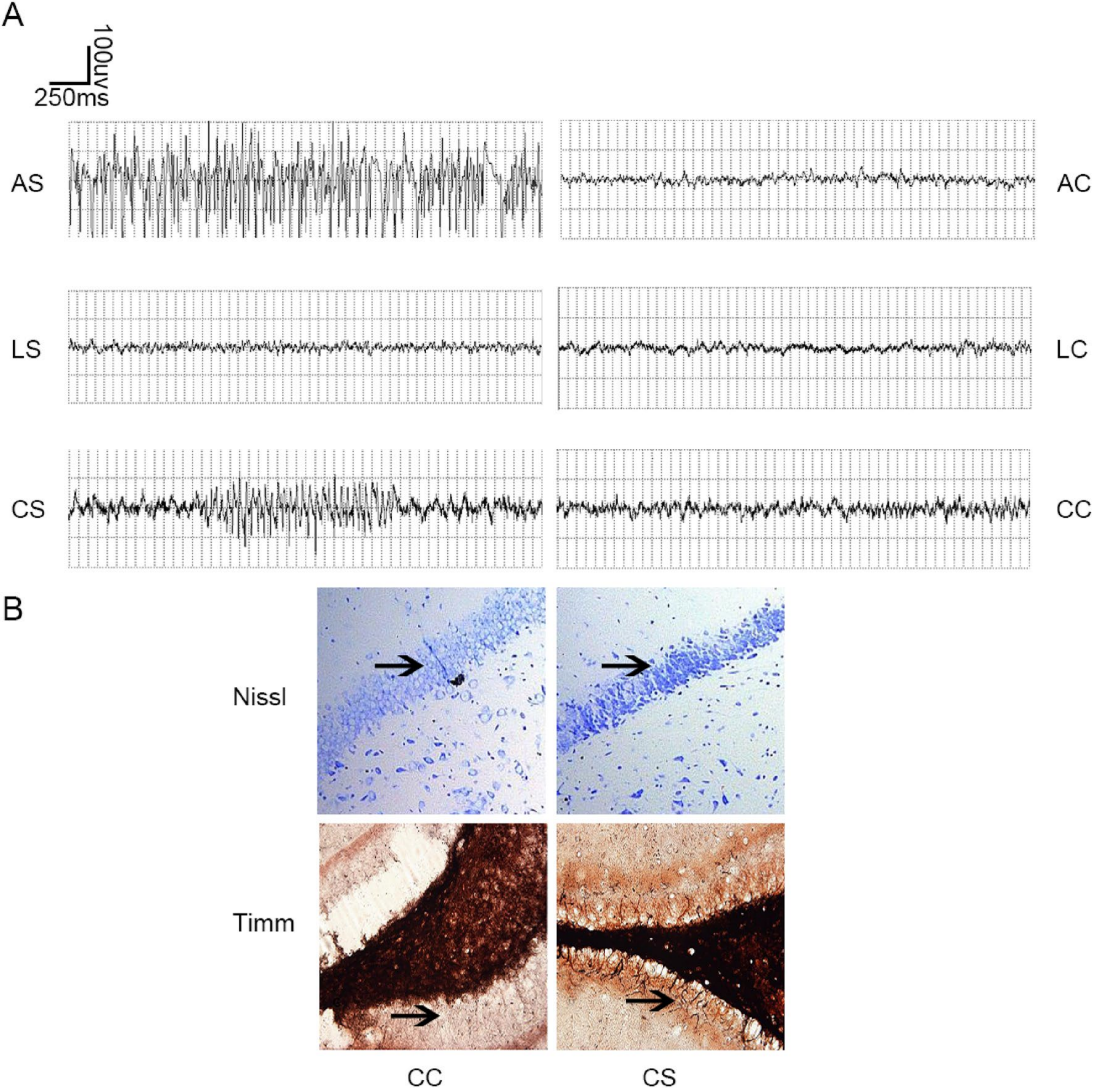
Construction of MTLE animal model was successful. (**A**) Electroencephalogram (EEG) results of the six groups of rats (n ≥ 3). (**B**) Nissl and Timm staining in the dentate gyrus (DG) region (×400).

After an intraperitoneal injection of pilocarpine for 10–25 min, the rats in the AS group showed SE, which was stopped with chloral hydrate. The rats in the CS group showed spontaneous seizures that were generally characterized by a focal onset (immobility, mechanical mastication, mouth clonus, forelimb clonus), occasionally culminating in a generalized convulsive stage lasting approximately 30 s–1.5 min, once to several times per day. Spontaneous seizures often occurred 3 weeks after SE and tended to stabilize 8 weeks after SE. These behavioural changes were consistent with the features of human MTLE. The EEG wave forms were monitored at different time points. A continuous epileptic spike wave form was detected in the AS group, while a discontinuous epileptic spike wave form was detected in the CS group. There was no difference in EEG wave forms between the LS and LC groups (Fig. 1A and supplementary materials).

### Neuronal loss and mossy fibre growth in the MTLE models

The hippocampal cytoarchitecture of the dentate gyrus (DG) at the chronic stage after pilocarpine was evaluated with Nissl and Timm staining (Fig. 1B). Regularly shaped neurons and abundant Nissl bodies were found in the DG region of the control groups. However, cell swelling and lysis, few Nissl bodies, and extensive cytoplasmic vacuolization appeared in the CS group. In this animal model, mossy fibres from granule cells in the DG region undergo reorganization of their terminal projections during the chronic stages of MTLE. In the CC group, rare black granules were observed in the DG region, but no obvious mossy fibre growth was observed; in the CS model group, moderate amounts of black particles were observed in the inner molecular layer, and obvious mossy fibre growth was observed in the inner molecular layer.

### The interaction between CLC-2 and NEDD4-2 in the MTLE rat hippocampus

**Figure 2:**
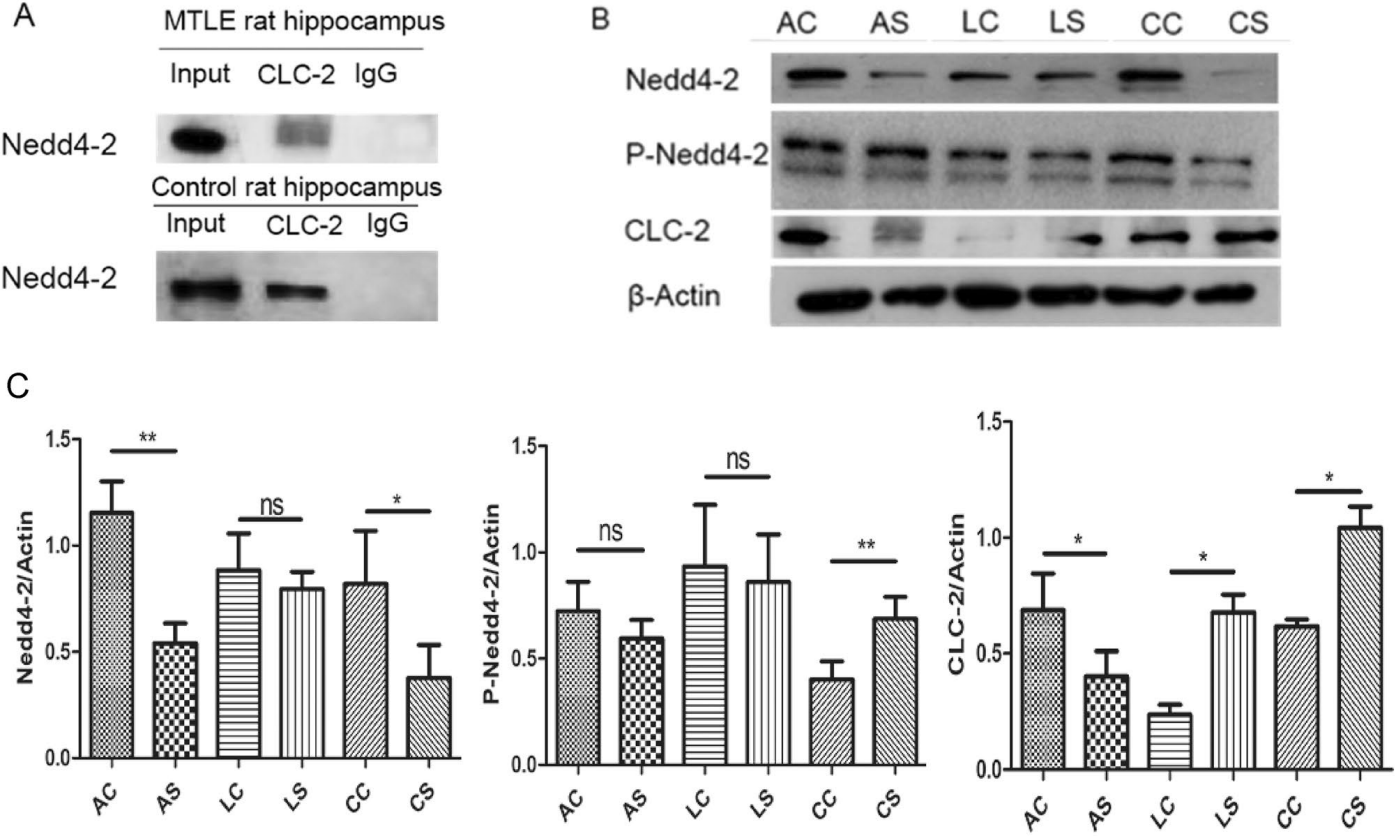
Decreased Nedd4-2 expression enhances its phosphorylation and increases the expression of the downstream substrate channel protein CLC-2 during MTLE development. (**A**) Coimmunoprecipitation analysis to examine the interaction between Nedd4-2 and CLC-2 in the MTLE and control rat hippocampus. (**B**) Western blotting analysis of Nedd4-2, P-Nedd4-2 and CLC-2 protein levels in MTLE and control rats. (**C**) Quantitative analysis of the result in (**B**) (n = 3) (error bars represent ± SEM). Band intensities of Nedd4-2, P-Nedd4-2 and CLC-2 were quantified and normalized to corresponding Actin immunoblotting signals by Image J. The intensities at time point 0 of each group were set as 100%. **p < 0.01, *p < 0.05, “ns” represents no statistical significance.

Co-IP confirmed the interactive relationship between **NEDD4-2** and CLC-2 in the MTLE and control rat hippocampus (Fig. 2A). Western blot analysis revealed the presence of CLC-2 in the buffer eluted from anti-NEDD4-2 G Sepharose beads, confirming that the interaction between CLC-2 and **NEDD4-2** in the MTLE rat hippocampus.

### Dynamic expression of NEDD4-2 and CLC-2 in the MTLE models

The expression levels of **NEDD4-2**, phosphorylated **NEDD4-2** (P- **NEDD4-2**) and CLC-2 in the hippocampi of MTLE rats were detected by western blot (Fig. 2B,C and supplementary materials). Compared to the control group, the protein levels of **NEDD4-2** in hippocampus of rat were lower in the AS and CS groups, and P- **NEDD4-2** expression was upregulated in the CS group, while CLC-2 expression was downregulated in the AS group and upregulated in the LS and CS groups. Thus, these results may suggest that NEDD4-2 protein levels may regulate the expression of the downstream substrate channel protein CLC-2 during the development of MTLE.

### NEDD4-2 regulates the protein levels of CLC-2 in primary hippocampal neurons

**Figure 3:**
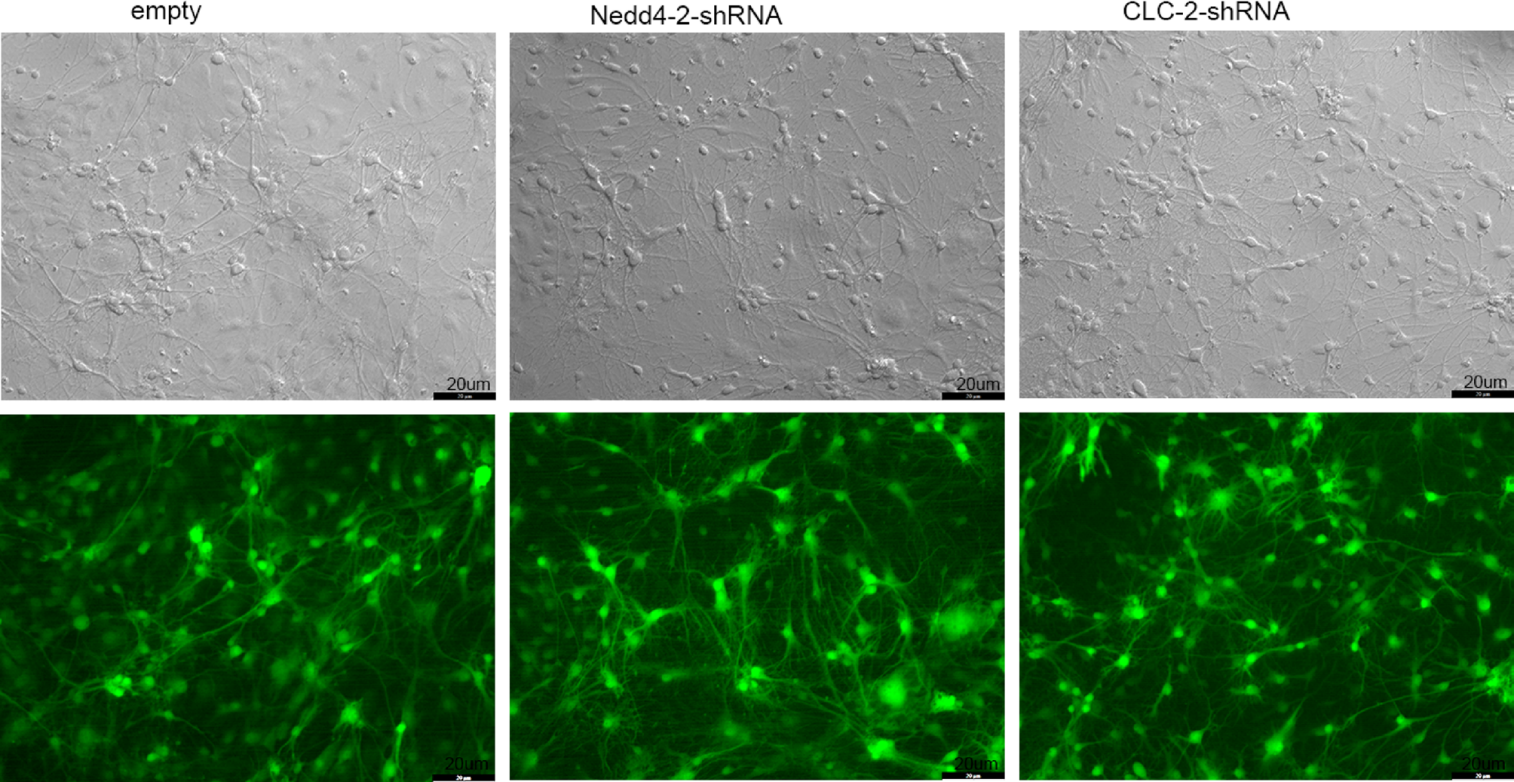
Green fluorescence from the lentivirus indicated a transfection efficiency of shRNA-Nedd4-2 and shRNA-CLC-2 in primary hippocampal neuron cells.

**Figure 4:**
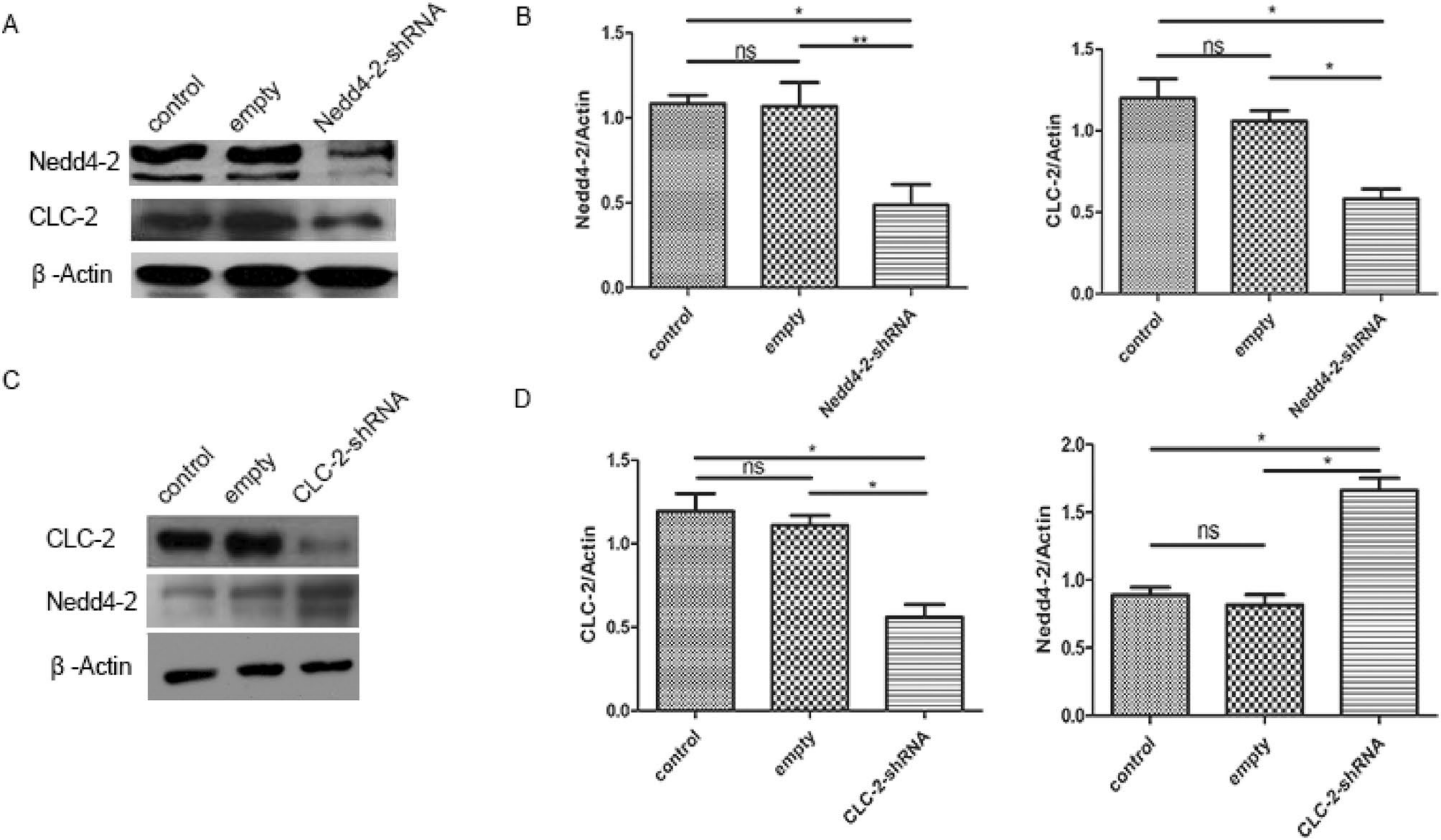
(**A**, **C**) Western blotting analysis of Nedd4-2 and CLC-2 protein levels after Nedd4-2 or CLC-2 was respectively knocked down by shRNA in primary hippocampal neuron cells. (**B**, **D**) Quantitative analysis of the result in (**A**, **C**), (n = 3) (error bars represent ± SEM). Band intensities of Nedd4-2 and CLC-2 were quantified and normalized to corresponding Actin immunoblotting signals by Image J. The intensities at time point 0 of each group were set as 100%. **p < 0.01, *p < 0.05, “ns” represents no statistical significance.

shRNA-**NEDD4-2**, shRNA-CLC-2 and control plasmids were transfected into primary hippocampal neurons by lentivirus infection, and analysis of green fluorescence from the lentivirus indicated a transfection efficiency greater than 90% (Fig. 3A). The knock-down efficiency of **NEDD4-2** and CLC-2 were detected by western blot shown as Fig. 4A,B and supplementary materials. Interesting, the protein levels of CLC-2 was significantly decreased in the **NEDD4-2**-shRNA groups, and **NEDD4-2** expression was significantly upregulated in the CLC-2-shRNA groups (Fig. 4C,D and supplementary materials). These results demonstrated that NEDD4-2 regulated the expression of CLC-2, and CLC-2 negatively regulated the protein levels of NEDD4-2.

### Effects of NEDD4-2 on neuronal discharge in the epileptic cell model

**Figure 5:**
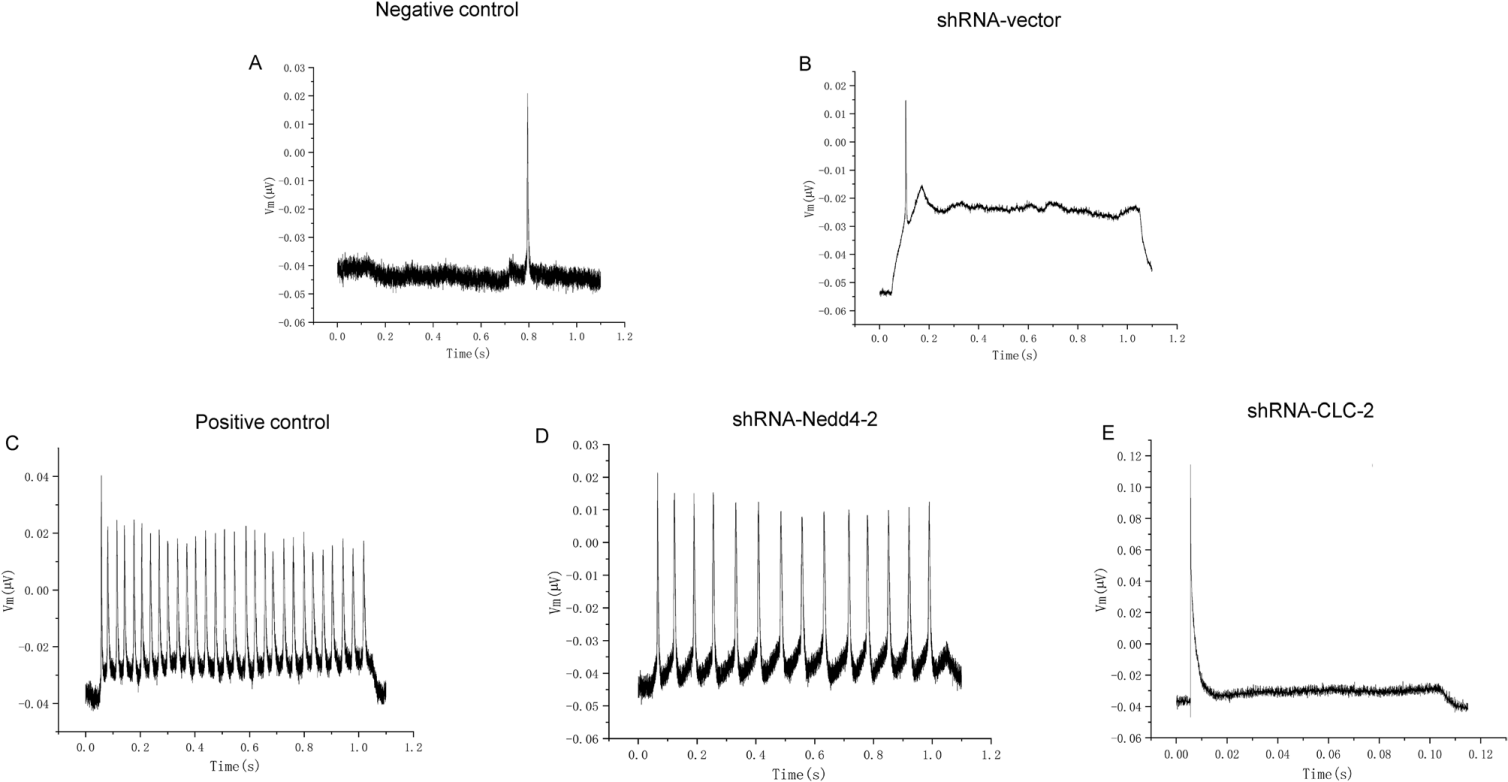
Nedd4-2 and CLC-2 affected neuronal firing. (**A**–**E**) Discharge of primary hippocampal neurons in each group with different treatment. (**A**) primary hippocampal neurons in physiological recording solution without MgCl^2^; (**B**) primary hippocampal neurons transfected with shRNA-vector in physiological recording solution without MgCl^2^; (**C**) primary hippocampal neurons in physiological recording solution with MgCl^2^; (**D**) primary hippocampal neurons transfected with shRNA-Nedd4-2 in physiological recording solution with MgCl^2^; (**E**) primary hippocampal neurons transfected with shRNA-CLC-2 in physiological recording solution with MgCl^2^.

Electrophysiological changes in primary hippocampal neurons after plasmid transfection were observed by patch-clamp electrophysiology. The discharges of the epileptic cells were significantly higher than that of the untreated controls. In the shRNA-NEDD4-2 and shRNA-CLC-2 groups, the frequency and amplitude of neuronal discharges decreased substantially, implying that NEDD4-2 and CLC-2 affected neuronal firing (Fig. 5A–E).

## Discussion

Many studies have shown that NEDD4-2 regulates the expression of ion channels and is involved in the pathogenesis of epilepsy^9,10,25–27^. CLC-2 is a member of the voltage-gated chloride channel family and a substrate protein of NEDD4-2^17^. Previous studies have also focused on the role of CLC-2 in the pathogenesis of epilepsy^28^. The role of CLC-2 in the pathogenesis of epilepsy has been controversial. The involvement of CLC-2 in epileptogenesis may be related to a reduction in intracellular chloride levels, thereby regulating the current and excitability of hippocampal pyramidal cells^18^. In CLC-2 knockout mice, increased inhibition seemingly balances the hyperexcitability of the network and thereby prevents epilepsy^29^. Here, our study utilized the MTLE model induced by lithium-pilocarpine in 21-day-old Sprague–Dawley rats and an epileptic cell model of hippocampal neurons from neonatal Sprague–Dawley rats. We utilized a combination of advanced research techniques, such as Co-IP, western blot, plasmid transfection and patch-clamp electrophysiology, to explore the role of the NEDD4-2/CLC-2 ubiquitin signalling pathway in MTLE pathogenesis.

In this study, MTLE was induced by lithium-pilocarpine injection in young rats, and their behaviour together with EEG recordings were observed to confirm the success of the MTLE induction. The behavioural and EEG results indicated that the observed pilocarpine-induced epilepsy in Sprague–Dawley rats was consistent with human MTLE disease. We also performed Nissl and Timm staining of the hippocampal tissues from MTLE model rats, which further confirmed that our MTLE animal model was successful.

Using the MTLE model induced by lithium chloride and pilocarpine, we confirmed the interaction between NEDD4-2 and CLC-2 by Co-IP. The protein levels of NEDD4-2 and CLC-2 were further analysed by western blot, which revealed dynamic expression levels of these proteins in the hippocampi of MTLE rats. Compared with its expression in the control group, the expression of NEDD4-2 was decreased in the AS and CS groups, but no significant difference in NEDD4-2 expression was found between the LS and LC groups. CLC-2 was downregulated in the AS group but upregulated in the LS and CS groups. These results indicate that the ubiquitin signalling pathway NEDD4-2/CLC-2 participates in the regulation of MTLE, with NEDD4-2 and CLC-2 playing different roles in the different developmental stages of MTLE. In the acute and chronic stage of MTLE, decreased expression of NEDD4-2 may regulate its phosphorylation form, however, further investigations would be needed to elucidate the associations between NEDD4-2 and phosphorylated NEDD4-2, although we did observe the change of protein levels of both NEDD4-2 and phosphorylated NEDD4-2.

To further confirm that NEDD4-2 regulates the firing of neurons by regulating CLC-2, we constructed an epileptic cell model from neonatal rat hippocampal neurons. NEDD4-2-shRNA, CLC-2-shRNA and empty plasmids were constructed and transfected into rat hippocampal neurons by lentivirus infection. The expression of CLC-2 was downregulated in the NEDD4-2-shRNA groups, clearly indicating that NEDD4-2 can regulate the expression of its downstream substrate CLC-2. CLC-2-shRNA and empty plasmids were also constructed and transfected into rat hippocampal neurons to examine the negative feedback effect of CLC-2 on NEDD4-2. NEDD4-2 expression was upregulated in the CLC-2-shRNA groups, suggesting that decreased CLC-2 activity stimulates NEDD4-2 overexpression. Importantly, patch-clamp electrophysiology was used to assess the electrophysiological changes in the epileptic cell model. The neuronal discharge frequency and amplitude were significantly decreased in the NEDD4-2shRNA groups, which indicated that NEDD4-2 affects the firing of neurons by regulating the expression of CLC-2. This result also successfully confirmed that NEDD4-2 and CLC-2 are involved in the pathogenesis of epilepsy and there is a negative feedback regulation relationship. The expression of CLC-2 is regulated by the upstream molecule NEDD4-2, which also explains the dual role of CLC-2 in the pathogenesis of epilepsy.

As we all know, multiple substrates of Nedd4 family members mediate presynaptic vesicle activity, including α-synuclein^30–32^ and tyrosine kinase A receptors^33–35^. In addition to CLC-2, other substrates of Nedd4-2 potentially involved in regulating neuronal activity, such as voltage-gated sodium channels Nav1.6 and voltage-gated potassium channels Kv7.3/KCNQ3^36–39^. Although our data showed that CLC-2 mediates Nedd4-2-associated neuronal discharge and seizures in MTLE rats, it does not rule out the potential contributions of other substrates of NEDD4-2. The expression levels, and subcellular distribution of Nedd4-2’s other substrates are pending further investigation. As we described previously, future studies are expected to elucidate the detailed functions and other substrates of Nedd4-2 in central nervous system, and provide better understanding of the mechanism underlying NEDD4-2 regulating epilepsy.

## Conclusion

We herein detected that NEDD4-2 is involved in the pathogenesis of MTLE via CLC-2 both in vivo and in vitro for the first time. NEDD4-2 and CLC-2 playing different roles in MTLE rat models at different stages. In the chronic stage of MTLE, decreased expression of NEDD4-2 enhances its phosphorylation and increases the expression of the downstream substrate channel protein CLC-2, while deletion of CLC-2 increased the NEDD4-2 expression, demonstrating a negative feedback regulation relationship between NEDD4-2 and CLC-2. This study provides a theoretical basis for the identification of new antiepileptic drug targets in the future (Supplementary information).

### Supplementary Information

Supplementary Video 1.

Supplementary Video 2.

Supplementary Video 3.

Supplementary Video 4.

Supplementary Information.

## Data Availability

The datasets used and/or analyzed during the current study available from the corresponding author on reasonable request.
