# Supplementary material for: NEDD4-2 and the CLC-2 channel regulate neuronal excitability in the pathogenesis of mesial temporal lobe epilepsy
Source: Sci Rep. 2024 Feb 28;14:4835. doi: 10.1038/s41598-024-52399-4 (PMC10902323; doi:10.1038/s41598-024-52399-4)
Supplement: Supplementary file 5 — Supplementary Information. [file 41598_2024_52399_MOESM5_ESM.pptx]

## Slide 1
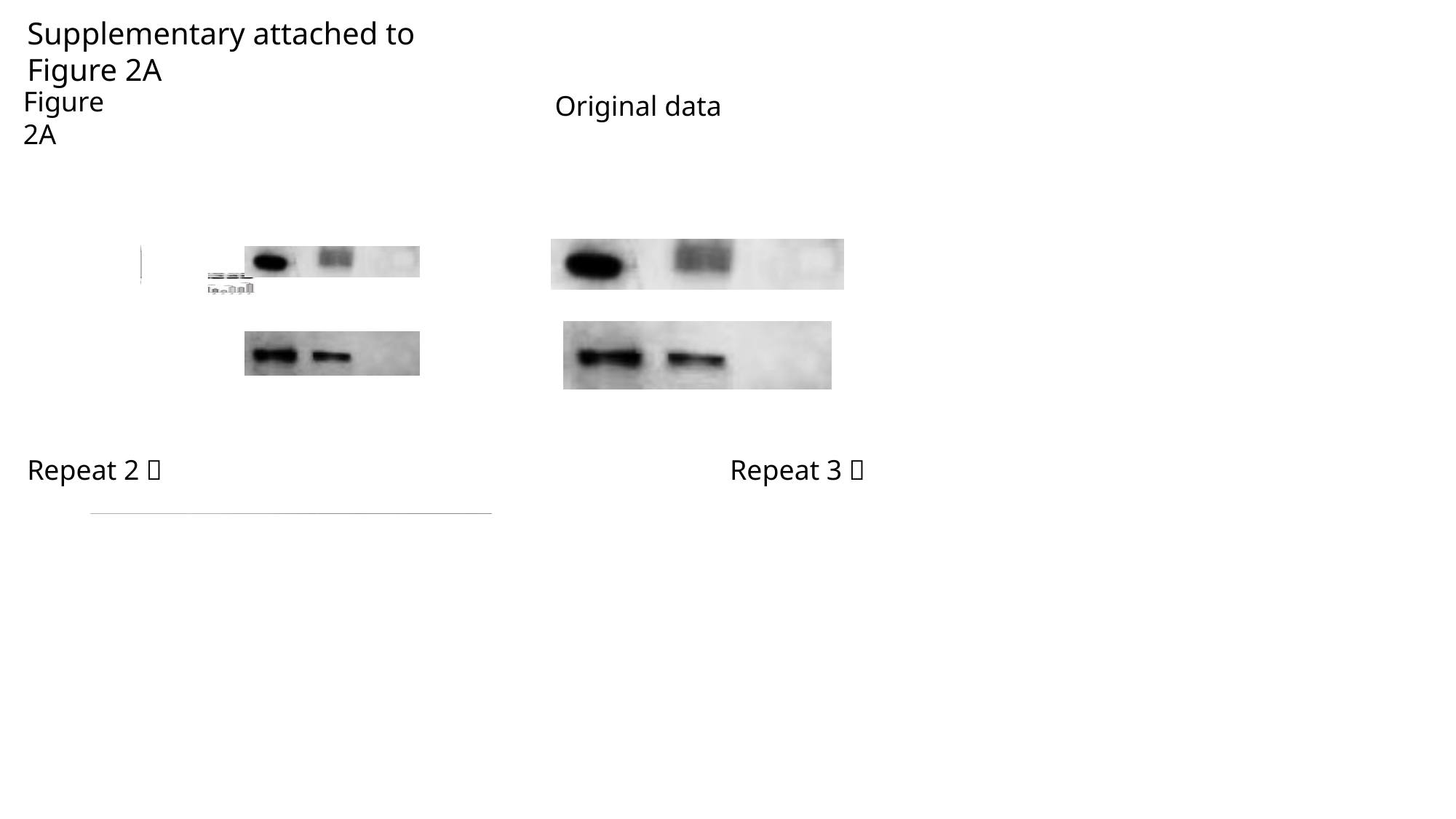

Supplementary attached to Figure 2A
Figure 2A
Original data
Repeat 2：
Repeat 3：

## Slide 2
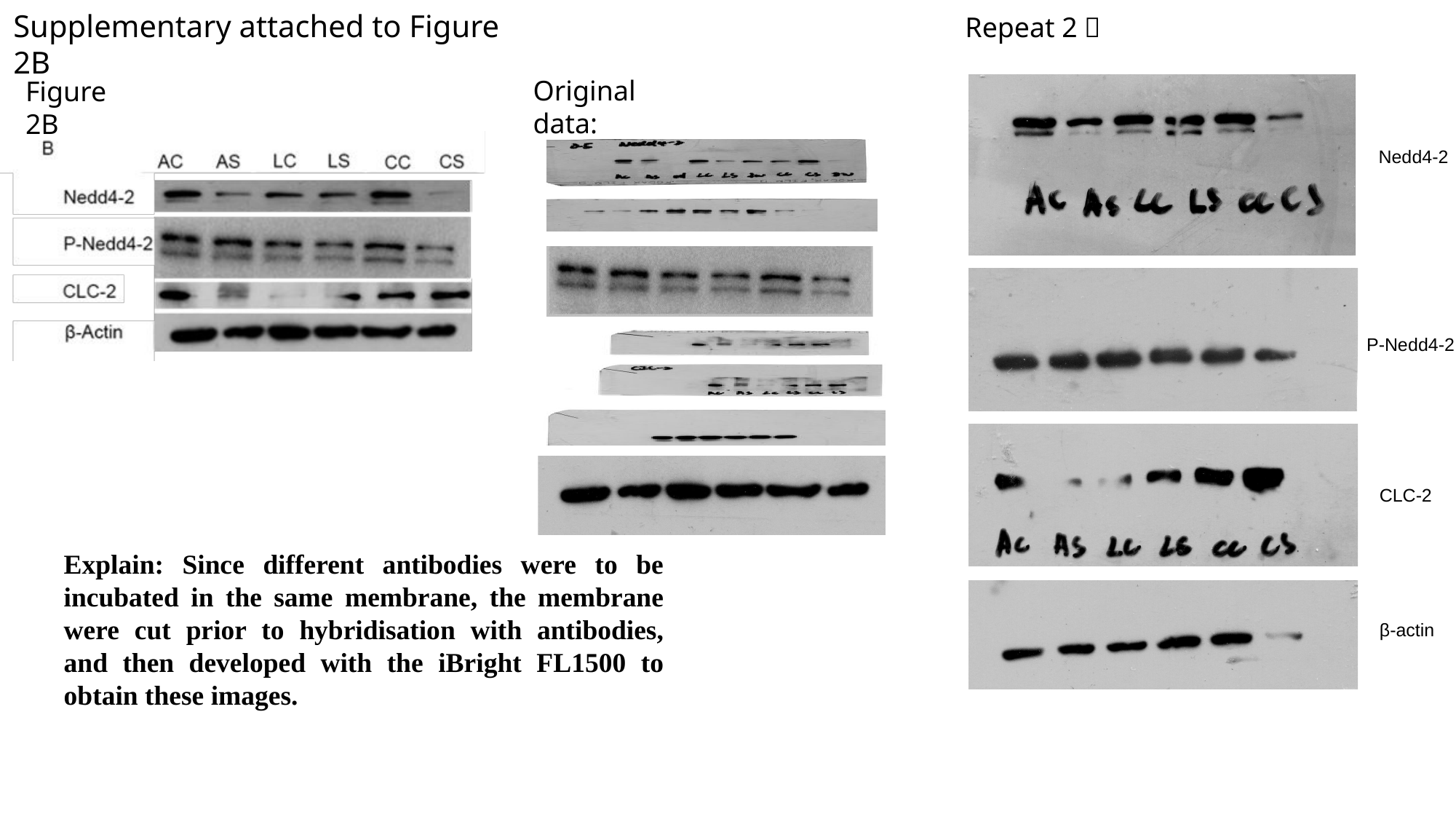

Supplementary attached to Figure 2B
Repeat 2：
Original data:
Figure 2B
Nedd4-2
P-Nedd4-2
CLC-2
Explain: Since different antibodies were to be incubated in the same membrane, the membrane were cut prior to hybridisation with antibodies, and then developed with the iBright FL1500 to obtain these images.
β-actin

## Slide 3
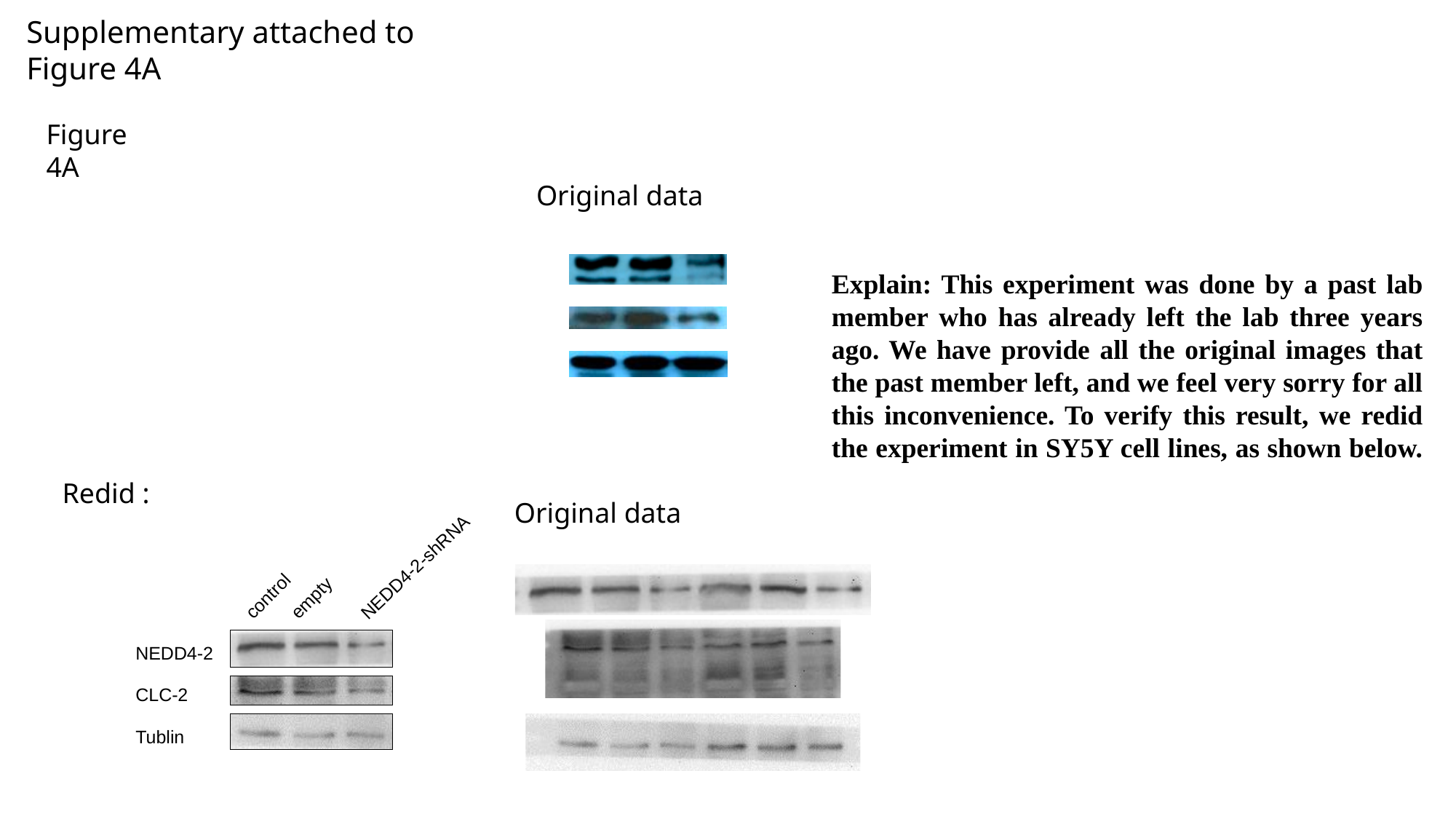

Supplementary attached to Figure 4A
Figure 4A
Original data
Explain: This experiment was done by a past lab member who has already left the lab three years ago. We have provide all the original images that the past member left, and we feel very sorry for all this inconvenience. To verify this result, we redid the experiment in SY5Y cell lines, as shown below.
Redid :
Original data
NEDD4-2-shRNA
control
empty
NEDD4-2
CLC-2
Tublin

## Slide 4
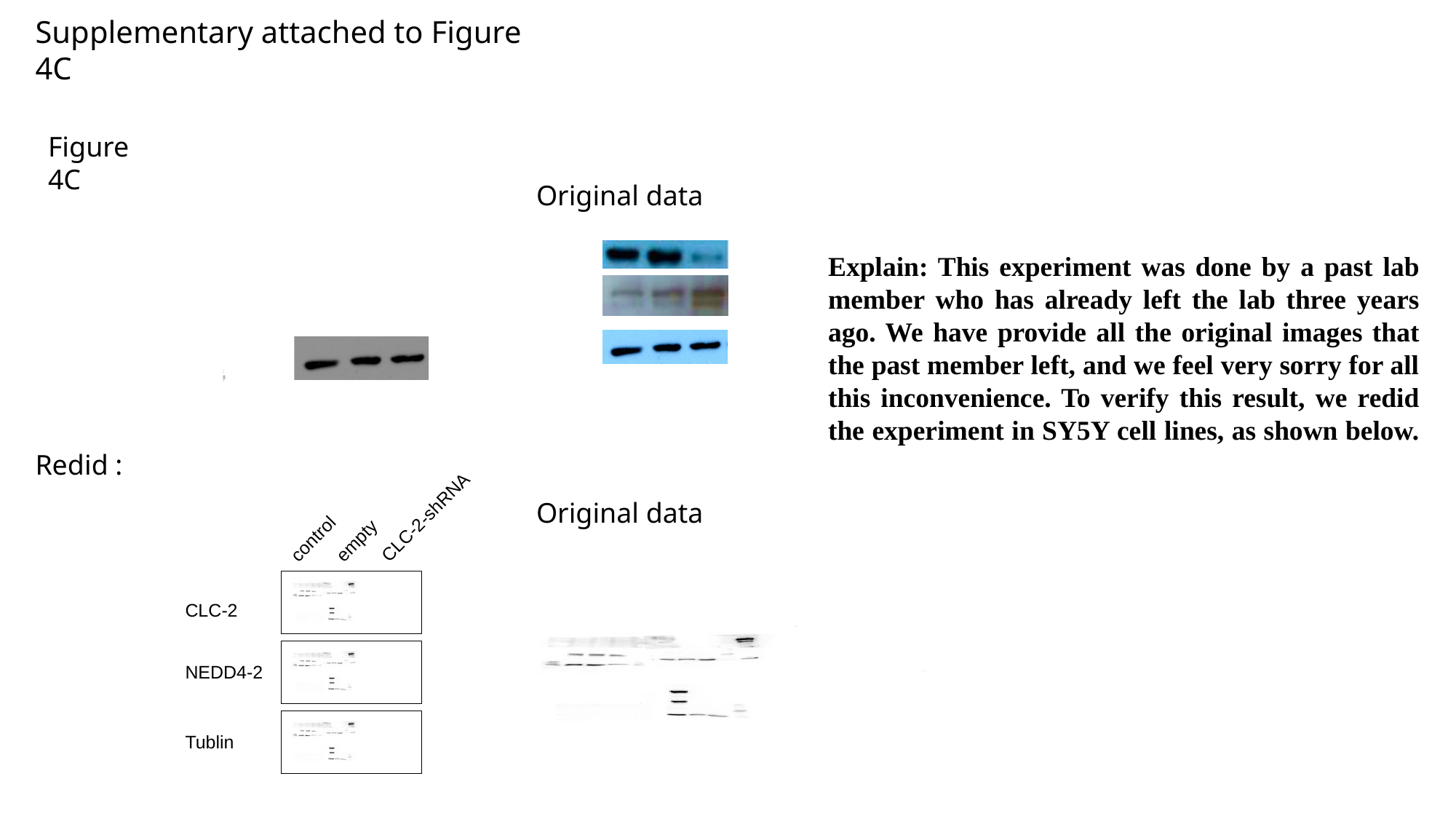

Supplementary attached to Figure 4C
Figure 4C
Original data
Explain: This experiment was done by a past lab member who has already left the lab three years ago. We have provide all the original images that the past member left, and we feel very sorry for all this inconvenience. To verify this result, we redid the experiment in SY5Y cell lines, as shown below.
control
empty
CLC-2-shRNA
CLC-2
NEDD4-2
Tublin
Redid :
Original data

## Slide 5
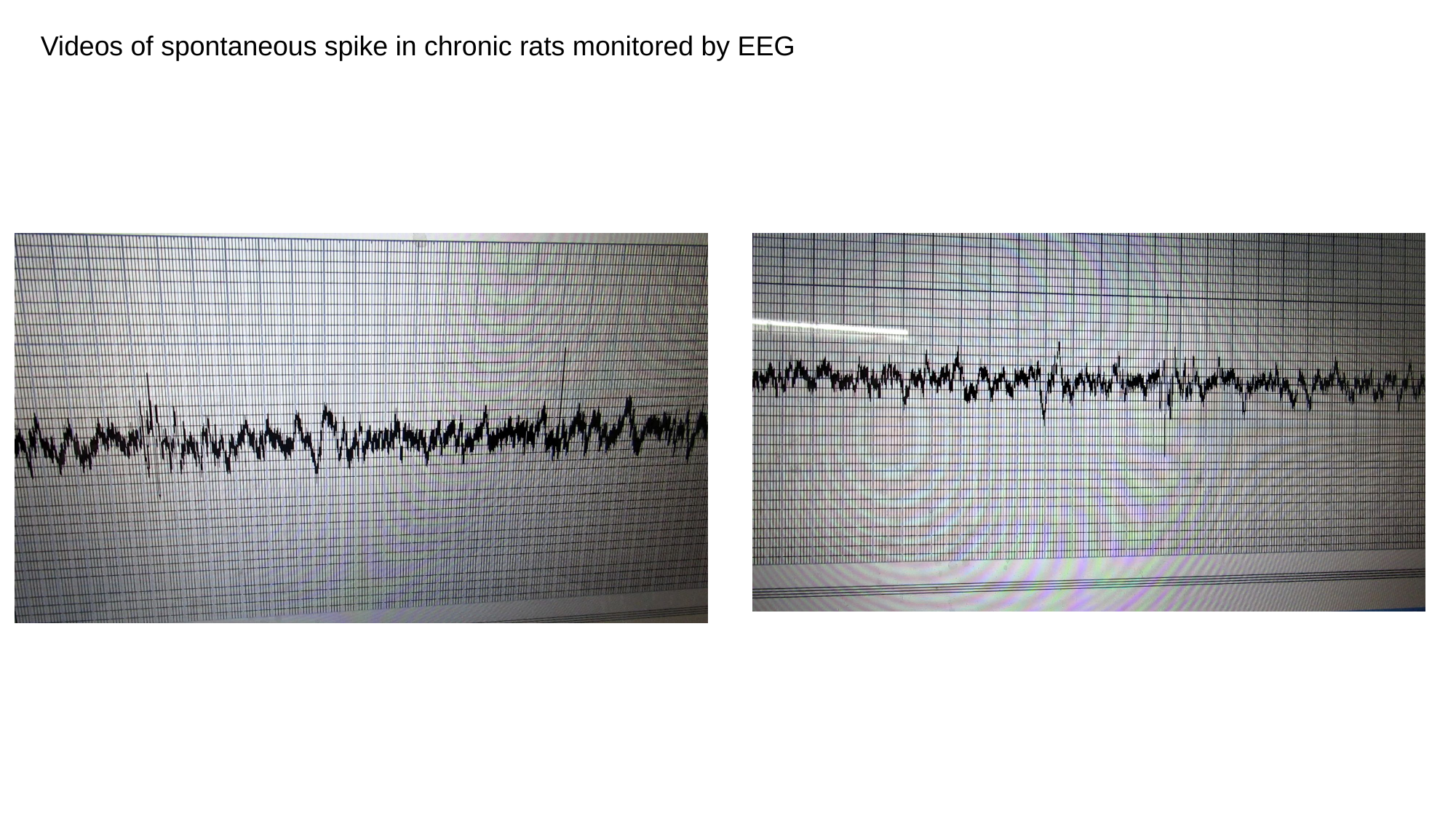

Videos of spontaneous spike in chronic rats monitored by EEG

## Slide 6
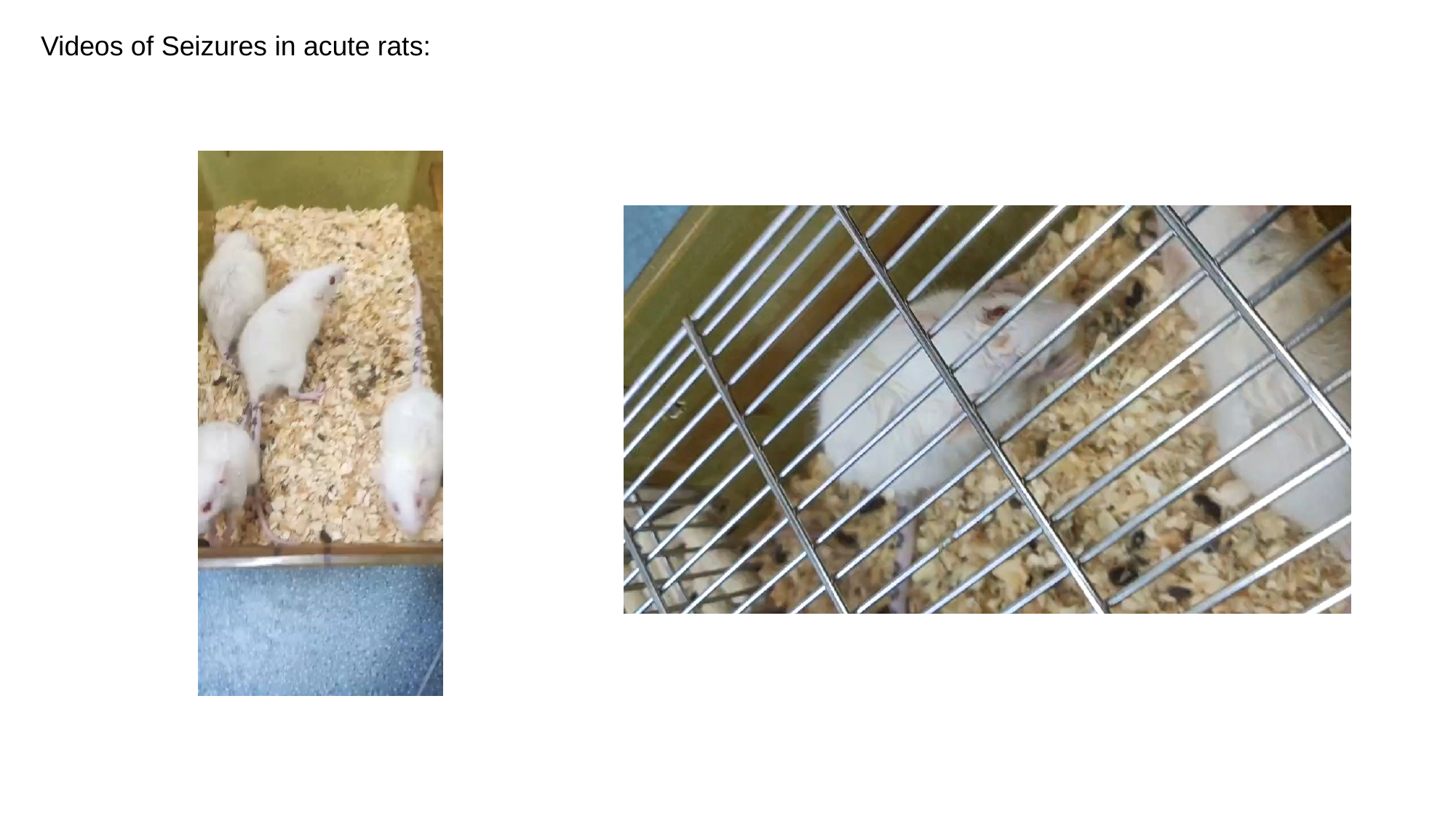

Videos of Seizures in acute rats:
